# Effect of Drought and Low P on Yield and Nutritional Content in Common Bean

**DOI:** 10.3389/fpls.2022.814325

**Published:** 2022-03-29

**Authors:** Millicent R. Smith, Eric Dinglasan, Erik Veneklaas, Jose Polania, Idupulapati M. Rao, Stephen E. Beebe, Andrew Merchant

**Affiliations:** ^1^School of Life and Environmental Sciences, Faculty of Science, The University of Sydney, Sydney, NSW, Australia; ^2^Queensland Alliance for Agriculture and Food Innovation, The University of Queensland, Saint Lucia, QLD, Australia; ^3^School of Biological Sciences and Institute of Agriculture, The University of Western Australia, Crawley, WA, Australia; ^4^Centro Internacional de Agricultura Tropical (CIAT), Cali, Colombia

**Keywords:** abiotic stress, legume, *Phaseolus*, phosphorus, protein, water deficit

## Abstract

Common bean (*Phaseolus vulgaris* L.) production in the tropics typically occurs in rainfed systems on marginal lands where yields are low, primarily as a consequence of drought and low phosphorus (P) availability in soil. This study aimed to investigate the physiological and chemical responses of 12 bush bean genotypes for adaptation to individual and combined stress factors of drought and low P availability. Water stress and P deficiency, both individually and combined, decreased seed weight and aboveground biomass by ∼80%. Water deficit and P deficiency decreased photosynthesis and stomatal conductance during plant development. Maximum rates of carboxylation, electron transport, and triose phosphate utilization were superior for two common bean genotypes (SEF60 and NCB226) that are better adapted to combined stress conditions of water deficit and low P compared to the commercial check (DOR390). In response to water deficit treatment, carbon isotope fractionation in the leaf tissue decreased at all developmental stages. Within the soluble leaf fraction, combined water deficit and low P, led to significant changes in the concentration of key nutrients and amino acids, whereas no impact was detected in the seed. Our results suggest that common bean genotypes have a degree of resilience in yield development, expressed in traits such as pod harvest index, and conservation of nutritional content in the seed. Further exploration of the chemical and physiological traits identified here will enhance the resilience of common bean production systems in the tropics.

## Introduction

Common bean (*Phaseolus vulgaris* L.) is the most important grain legume for human consumption (Beebe, 2012). In tropical regions where common bean is typically cultivated, 60% of production is at risk of intermittent or terminal drought stress (Beebe et al., 2008) and 50% of the area suffers from low phosphorus (P) availability in soil (Beebe et al., 2009). Consequently, drought frequently occurs in combination with low P availability (Ho et al., 2005) in regions where producers have less capital investment for improvements (Broughton et al., 2003; Cavalieri et al., 2011). In common bean, drought has been demonstrated to significantly impact yield quantity as a consequence of interference with pod development and seed fill (Beebe et al., 2008) and disruption of processes related to carbon partitioning (Cuellar-Ortiz et al., 2008). Low P availability also reduces the yield of common bean by impacting mainly on photosynthesis, metabolism, and carbohydrate partitioning between source and sink tissues (Nielsen et al., 2001; Hermans et al., 2006). Chemical and physiological acclimation and adaptation to the effects of abiotic stress are multifaceted and likely associated with greater overall plant efficiency allowing the plant to adapt to a range of abiotic stresses (Beebe et al., 2008). For example, physiological and chemical responses such as the remobilization of carbohydrates into reproductive tissues are a shared mechanism for resistance, at least to drought and limited P availability within common bean (Beebe et al., 2008; Cuellar-Ortiz et al., 2008). Understanding the physiological processes that underlie responses to abiotic stress and the implications of this on yield and nutritional quality is critical to identify superior common bean genotypes for production in tropical regions.

The impact of drought and low P availability on common bean yield is well described. What is less known is the impact of these resource limitations on the nutritional quality of the resulting seed. In particular, while we know that the recycling of nutrients between plant pools is essential for producing seeds with high concentrations of proteins, lipids, and starch (Bennett et al., 2011), less known is the physiological and structural adaptations to the combined effects of abiotic stress such as drought and low P on the overall nutritional quality of seed. The soluble components of leaf tissue represent the transportable pool of resources that turns over rapidly alongside less-labile components of storage and structural material, and these pools, therefore, hold potential for indicating short-term resource limitations.

To investigate the effects of both water and P deficit in source and sink tissues of common bean, a replicated study was conducted to investigate the genotypic differences in bred genotypes of common bean to an individual and combined water deficit and P deficiency on seed yield and nutritional quality. Specifically, we tested the following hypotheses: (1) variation in the magnitude of yield decrease will be observed among genotypes and treatment combinations, (2) concomitant changes in the content and concentration of nutrients (mineral nutrients, amino acids) will be observed on a background of reductions in yield due to treatment effects and (3) qualitative and quantitative changes in nutrient content and concentration in seeds will be reflected in foliar nutrient content.

## Materials and Methods

### Experimental Design

Plants were established as a randomized complete block design with four treatments (main plots) and 12 genotypes as subplots with three replications. Treatments consisted of (i) low phosphorus + well-watered (LPWW), (ii) low phosphorus + water deficit (LPWD), (iii) high phosphorus + well-watered (HPWW), and (iv) high phosphorus + water deficit (HPWD). The low P treatment corresponded to a soil P application rate of 10 kg/ha, high P corresponded to a soil P application rate of 40 kg/ha (Polania et al., 2009) well-watered denotes plants watered to 80% of field capacity, and water deficit denotes suspension of irrigation at 15 days after sowing (DAS).

### Plant Material

A total of 12 bush bean genotypes belonging to the Middle American gene pool previously bred by the Centro Internacional de Agricultura Tropical (CIAT) and its partners were selected for their inclusion in the trial based on their tolerance to abiotic stress or commercial availability (see Table 1). Genotypes selected included superior drought and low P tolerant (NCB226, SEF60, SEN56, BFS35, BFS81), drought tolerant (SEF71, RCB593), low P tolerant (SEF73, Carioca, SXB412), and commercial checks (DOR390, Tio Canela) as previously described in Beebe et al. (2008) and Polania et al. (2016c). Pedigrees of drought, and drought- and low P–adapted lines used in this study included SEA 15, which is a progeny of SEA 5, which in turn was derived from an interracial cross including races Durango and Mesoamerica of the Middle American gene pool (Terán and Singh, 2002). SEA 15 also has Apetito (G 1759), a race Durango landrace, in its pedigree (Beebe et al., 2008). Seed color and growth habits of the genotypes varied (see Table 1). Growth habits used in this study, as described by Singh (1981) were previously classified as, 2A: an indeterminate growth habit lacking climbing ability, 2B: an indeterminate growth habit possessing some climbing ability, and 3B: an indeterminate growth habit with long main stem guide possessing moderate climbing ability. Some of the genotypes had been characterized previously under drought conditions (see Polania et al., 2016a,c).

**TABLE 1:** Description of 12 common bean genotypes included in the lysimeter trial at CIAT grown under individual and combined treatments of low phosphorus and water deficit conditions.

Objective	Genotype	Seed color	Growth habit
Superior drought and low P tolerant	NCB226	Black	2B
	SEF60	Red	2A
	SEN56	Black	2A
	BFS35	Red	2A
	BFS81	Red	2B
Drought tolerant	SEF71	Red	2A
	RCB593	Red	2B
Low P tolerant	SEF73	Red	2B
	Carioca	Cream striped	3B
	SXB412	Cream	2B
Commercial checks	DOR390	Black	2B
	Tio Canela	Red	2A

*Growth habit classification is explained in the text.*

### Experimental Site and Lysimeter Conditions

Plants were grown in soil lysimeters under a movable rainout shelter from September to December 2015, at the experimental station of the International Center for Tropical Agriculture (CIAT) in Palmira, Colombia, located at 3° 29″ N latitude, 76° 21″ W longitude, and an altitude of 965 m. Lysimeters were constructed from plastic transparent cylinders inserted into PVC pipes with a diameter of 20 cm and height of 120 cm (Polania et al., 2017). Lysimeters were filled with 60 kg of dry soil with the top 10 cm of the lysimeter left empty. The soil was collected from the CIAT field station in Darien, Colombia, located at 3° 53″ N latitude, 76° 31″ W longitude, and an altitude of 1,460 m. The bulk density of the soil used in the lysimeters was adjusted to 1.1 g/cm^3^ to facilitate root growth and drainage. The soil is described as an Andisol and is characterized as deficient in available P (Beebe et al., 2008). Available P content was determined (Bray-II) to be 5.7 mg/kg. Available P content was determined (Bray-II) to be 7.1 mg/kg in the low P treatment and 12.5 mg/kg in the high P treatment. For the duration of the trial, mean maximum and minimum air temperatures were 31.1°C and 20.1°C, respectively, with an average relative humidity (RH) of 58.4%. The initial soil moisture content of all four treatments was at 80% field capacity. Plants in the well-watered treatment were maintained at 80% field capacity by weighing the lysimeter two times each week, irrigating the top of the lysimeter and registering soil water content. Plants in the water deficit treatment were weighed two times each week to calculate the loss of soil moisture content. At harvest, the soil moisture content in the terminal drought treatments was determined to be 39% field capacity for high P (HPWD), and 42% of field capacity for low P (LPWD). The trial was managed with weeding and spraying of insecticides and fungicides as required.

### Leaf Gas Exchange Measurements

Gas exchange for each plant was measured using an LI-COR 6400 XT infra-red gas analyzer (LI-COR, Lincoln, NE, United States) for leaf-level photosynthesis, stomatal conductance, and sub-stomatal CO_2_ concentration at two growth stages, flowering (DAS 32–37) and mid-pod fill (DAS 44–47). Instantaneous leaf gas exchange measurements were made between the hours of 10:00 am and 2:00 pm. A fully expanded, non-shaded leaf was chosen for measurement at each time point. For spot measurements, conditions in the chamber were set to photosynthetically active radiation (PAR) of 1,200 μmol/m^2^/s, temperature maintained at 27°C and RH values were kept within the range of 65–75%. The CO_2_ mole fraction of reference air in the measuring chamber was set at 400 μmol/mol for ambient measurements and 2,000 μmol/mol for maximum rate of photosynthesis (*A*_*max*_) measurements. Spot measurements were completed within 5 days for each growth stage, with genotypes measured in order from earliest to latest flowering.

Relationships between net photosynthesis and sub-stomatal CO_2_ concentration (*A/c_*i*_* curves) were completed using the automated program on the LI-COR 6400 XT. Parameters were set such that *c*_*a*_ be stepped down from 400 to 50 μmol/mol before moving back up from 400 to 2,000 μmol/mol. Conditions in the chamber were set to a PAR of 1,200 μmol/m^2^/s, leaf temperature was maintained at 27°C and RH values were kept within the range of 65–75%. *A/c_*i*_* curves were carried out on three genotypes; DOR390, NCB226, and SEF60 under low and high P treatments for well-watered plants only (i.e., no combined water deficit treatment). *A/c_*i*_* curves were taken at the same growth stages as mentioned earlier, flowering and mid-pod fill. Parameters that can limit maximum rates of photosynthesis; maximum rates of carboxylation (*V*_*cmax*_), electron transport (*J*), and triose phosphate utilization (TPU) were derived from version 2.0 of the *A/c_*i*_* curve fitting calculator described by Sharkey (2015).

### Tissue Collection

Following gas exchange measurements, one fully expanded leaf not measured in the LI-COR6400 chamber was collected from each plant using a razor blade. The leaf sample was microwaved for 10 s using a conventional 900 W microwave oven to prevent metabolic activity according to the method outlined by Popp et al. (1996) and placed in an oven to dry at 65°C. At harvest, leaf area (LI-COR model LI-3000) and shoot biomass distribution (leaves, dead leaves, stems, pod walls, seed) were recorded. Shoot dry weight was determined after the shoot samples were dried in an oven at 65°C for 48 h. Harvest index and pod harvest index were determined by Equations 1, 2, respectively.


(1) $$Harvestindex=\frac{seedbiomassdryweightatharvest}{abovegroundplantbiomassdryweightatharvest}\times 100$$

(2) $$Podharvestindex=\frac{seedbiomassdryweightatharvest}{podbiomassdryweightatharvest}\times 100$$

### Extractions of Leaf and Seed Material

Samples of leaves and seeds were oven-dried at 65°C and ground using an oscillating matrix mill. Approximately 40 mg of ground leaf/seed sample was then weighed into a 2-mL microtube and extracted in a hot water mix according to the protocol outlined by Merchant et al. (2006). An additional 20 mg of ground seed material was placed with 1 ml of 6 M hydrochloric acid in a vacuum hydrolysis tube and digested for 24 h at 110°C. Extracts of the hot water extraction and digestion process were stored frozen at −80°C awaiting further analysis as described in the followings sections.

### Analysis of Carbon and Oxygen Isotope Abundance

Determination of carbon and oxygen isotope abundance on ground bulk samples of leaves and seed was completed using a Delta V Advantage isotope ratio mass spectrometer (IRMS) (Thermo Electron) with a Conflo IV interface (ThermoFisher Scientific, Bremen, Germany).

### Analysis of Plant Material for Amino Acids and Mineral Nutrients

Determination of soluble amino acids and total amino acids in extracted and digested samples, respectively, was completed using high-performance liquid chromatography (HPLC) coupled to a quadrupole time-of-flight mass spectrometer. HPLC separation was completed on an Agilent 1290 Infinity system (Agilent, Walbronn, Germany) using a Zorbax StableBond SB-CB18 column (150 mm × 2.1 mm, 3.5 μm, Agilent) including degasser, binary pump, temperature-controlled autosampler (maintained at 4°C), and column compartment (maintained at 30°C). The mobile phase was composed of water containing 0.1% formic acid (solution A) and methanol containing 0.1% formic acid (solution B). The flow rate was 0.3 mL/min with a gradient elution of 0–100% solution B, over 23 min for positive mode, respectively. Amino acids were detected by a quadrupole-time-of-flight mass spectrometer (Agilent 6520 accurate-mass) with a dual electrospray ionization (ESI) source. The mass spectrometer was operated with a full scan in positive FT mode for amino acid analysis. ESI capillary voltage was set at 4,000 V (+) ion mode and 3,500 V (−) ion mode and fragmentor at 135 V. The liquid nebulizer was set to 30 psig and the N drying gas was set to a flow rate of 10 L/min. The drying gas temperature was maintained at 300°C. Internal reference ions were used to continuously maintain mass accuracy. Molecular ions [(M+H)+ for amino acids] were extracted from the full scan chromatograms and peak areas integrated using Agilent MassHunter Workstation software (Agilent Technologies, Santa Clara, CA, United States).

Determination of soluble and total mineral nutrients in the extracted and digested samples, respectively, was completed using an inductively coupled plasma optical emission spectrometer (Varian Vista, Agilent Technologies, Santa Clara, CA, United States). Samples were prepared with a dilution of 400 μl of supernatant in 10 ml of ultra-pure Milli-Q water. Any results lower than the detection limit of the instrument were adjusted to zero.

### Statistical Analysis

Analysis was conducted in R software (R Core Team, 2021). Analysis of variance was performed wherein models were fitted using the lm function, where genotypes and treatments were treated as fixed effects. Tukey’s honest significant difference (HSD) test was used for multiple comparisons of means. Correlations and principal component analysis (PCA) were conducted to determine the relationships of yield and photosynthetic parameters. All figures were generated and visualized in R using ggplot2 and complimentary grammar of graphics packages.

## Results

### Water Deficit and Low P Availability Significantly Decrease Yield

**FIGURE 1:**
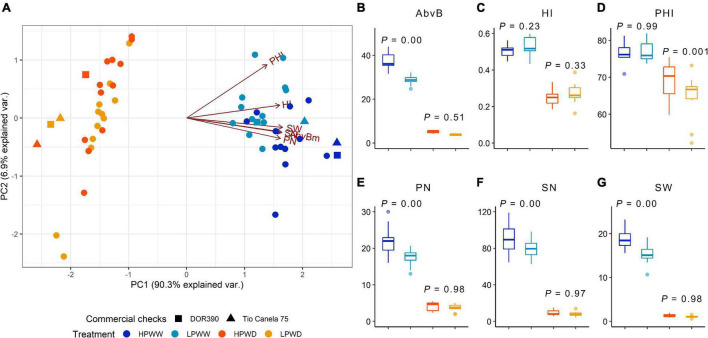
**(A)** Principal component analysis of yield parameters; pod harvest index (PHI), harvest index (HI), seed weight (SW, g), seed number (SN), aboveground biomass (AbvB, g), and pod number (PN) for 12 common bean genotypes under treatments of high phosphorus well-watered (HPWW; blue), low phosphorus well-watered (LPWW; light blue), high phosphorus water deficit (HPWD; orange), and low phosphorus water deficit (LPWD; yellow-orange). Commercial checks are illustrated by square and triangle shapes. **(B)** Aboveground biomass, **(C)** harvest index (HI), **(D)** pod harvest index (PHI), **(E)** pod number (PN), **(F)** seed number (SN), **(G)** seed weight (SW, g) for 12 common bean genotypes under treatments of high phosphorus well-watered (HPWW; blue), low phosphorus well-watered (LPWW; light blue), high phosphorus water deficit (HPWD; orange), and low phosphorus water deficit (LPWD; yellow-orange).

**FIGURE 2:**
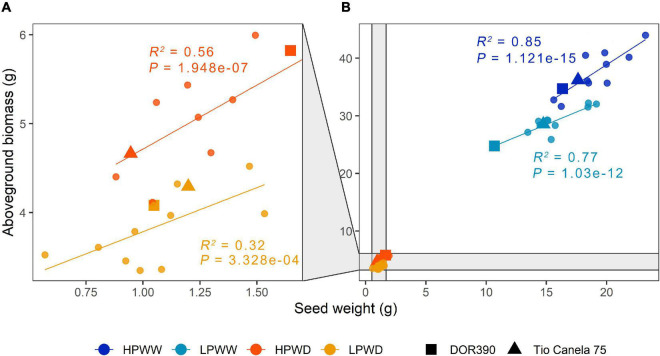
Seed weight (g) and aboveground biomass (g) for 12 common bean lines under different treatments. **(A)** High phosphorus water deficit (HPWD; orange) and low phosphorus water deficit (LPWD; light orange). **(B)** High phosphorus well-watered (HPWW; blue) and low phosphorus well-watered (LPWW; light blue). Standard errors have been removed for clarity (*n* = 3). Linear regression to calculate adjusted *R*^2^ and *P* values grouped according to treatment. Commercial checks are illustrated by square and triangle shapes.

About 97% of the cumulative variation among the different genotypes was explained by PC1 and PC2, wherein overall response to water deficit and low P availability significantly decreased yield (Figure 1). Overall, aboveground biomass, harvest index, pod number, seed number, and seed weight were strongly correlated with one another (*r* = 0.992–0.837; *P* = 4.44e-16–0.000), whereas pod harvest index relatively differed from the other parameters (*r* = 0.760–0.695; *P* = 4.44e-08–3.88e-10 (Figure 1A and Supplementary Table 1). Under well-watered (WW) conditions, P availability had a significant effect on all traits (aboveground biomass, *P* = 0.00; pod number, *P* = 0.00; seed number, *P* = 0.00; seed weight, *P* = 0.00) except harvest index (*P* = 0.23) and pod harvest index (*P* = 0.99) (Figures 1B–G). In contrast, under water deficit (WD) conditions, only pod harvest index (*P* = 0.001) was significantly affected by P availability (Figure 1D). All treatment comparisons between different yield parameters are summarized in Supplementary Table 2. Among yield parameters mentioned above, aboveground biomass and seed weight were strongly correlated with each other (*r* = 0.992; *P* = 0.000) (Figure 1). Increased seed weight was linearly proportional to high aboveground biomass, which was more prominent under well-watered (WW) conditions (HPWW/LPWW, *R*^2^ = 0.77–0.85) (Figure 2). In general, superior genotypes bred for resilience to abiotic stress did perform well under all treatments compared to the commercial checks DOR390 and Tio Canela (Figures 1A, 2).

### Photosynthesis and Stomatal Conductance Are Primarily Impacted by Water Deficit

**FIGURE 3:**
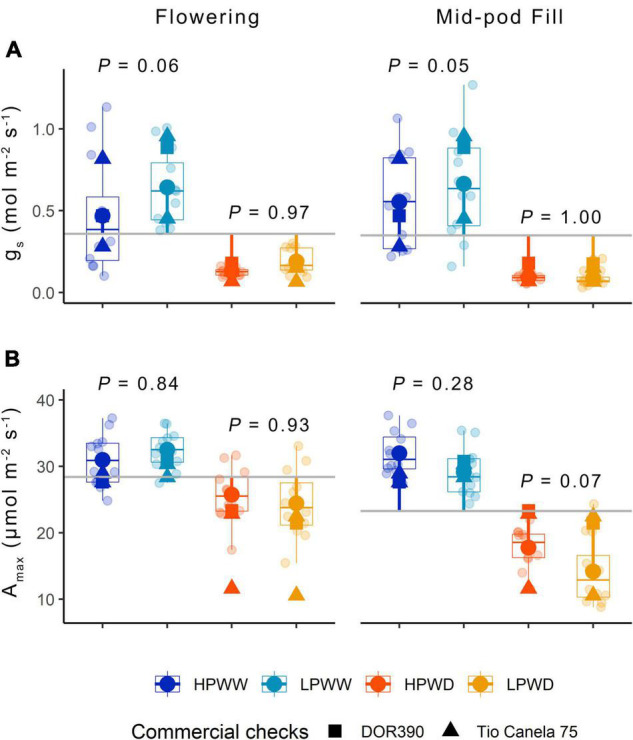
**(A)** Stomatal conductance *g*_*s*_ (mol/m^2^/s) and **(B)** *A*_*max*_ (μmol/m^2^/s) for 12 common bean genotypes under treatments of low phosphorus well-watered (LPWW), high phosphorus well-watered (HPWW), low phosphorus water deficit (LPWD). and high phosphorus water deficit (HPWD) at flowering and mid-pod fill stages. *Gray horizontal lines* represent overall means across the different treatments; *segment lines* represent the mean difference of individual treatment relative to the overall means. *P* values are according to treatment means comparison calculated using HSD test at ɑ = 0.05. Commercial checks are illustrated by square and triangle shapes. Multiple comparisons between treatments is provided in Supplementary Table 4.

Leaf-level gas exchange at both ambient [CO_2_] (data not shown) and maximum [CO_2_] shows a significant reduction in leaf-level photosynthesis in response to WD compared to plants grown under WW conditions (Figure 3). Corresponding reductions in stomatal conductance (*g*_*s*_) were observed with less than half the stomatal conductance for the WD treatment compared to the WW treatment (Figure 3). P supply impacted ambient rates of stomatal conductance under the WW treatment only (*P* = 0.06) (Figure 3). Ambient and maximum photosynthesis and stomatal conductance declined over development with statistically significant differences found between flowering compared to mid-pod fill (Supplementary Table 4). Ambient and maximum photosynthesis and stomatal conductance varied with genotype (Figure 3) with bred genotypes having greater instantaneous water use efficiency (higher photosynthetic capacity and lower stomatal conductance) under treatment conditions (Figure 3). Significant differences were also observed for maximum rates of carboxylation (*V*_*cmax*_), electron transport (*J*), and triose phosphate utilization (TPU) for genotypes SEF60 and NCB226 compared to the commercial check DOR390 (*P* < 0.05), suggesting an increased capacity of superior-bred genotypes to photoassimilate under low P supply than the commercially available check (see Supplementary Figure 1).

### Patterns in Isotope Abundance (δ^13^C and δ^18^O) Indicate the Severity of Water Deficit

**FIGURE 4:**
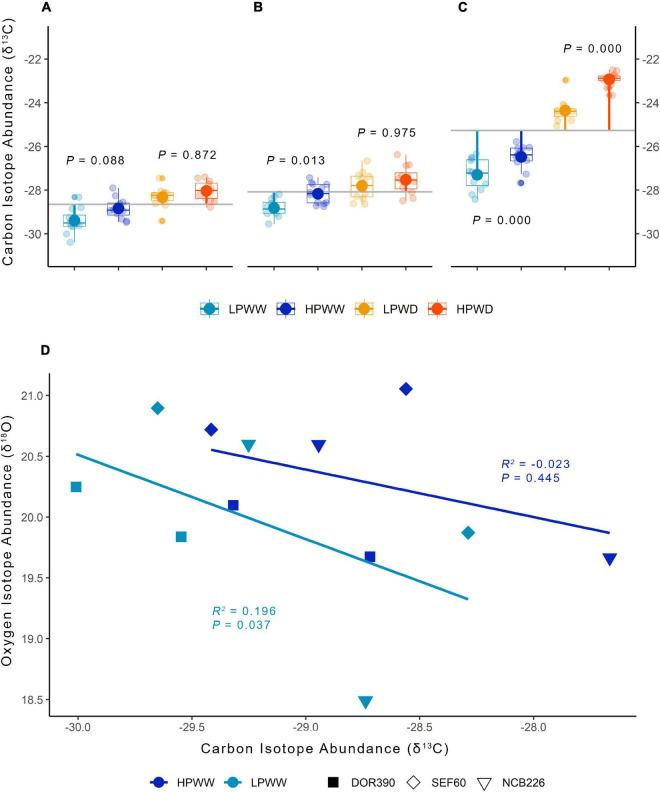
Carbon isotope abundance (δ^13^C) for bulk leaf tissue at flowering **(A)**, mid-pod fill **(B)**, and seed at harvest **(C)** for 12 common bean genotypes under treatments of low phosphorus well-watered (light blue), high phosphorus well-watered (blue), low phosphorus water deficit (yellow-orange), and high phosphorus water deficit (orange). The *gray line* represents the overall means across treatments at different development stages while *segment lines* represent the means per treatment showing the deviations from the overall means within the development stage. *P* values are according to treatment mean comparisons within the development stage calculated using the HSD test at ɑ = 0.05. Multiple comparisons between treatments are provided in Supplementary Table 5. **(D)** Carbon isotope abundance (δ^13^C) and oxygen isotope abundance (δ^18^O) for bulk leaf samples under treatments of low phosphorus well-watered (light blue) and high phosphorus well-watered (blue) for selected lines DOR390 (*filled square*), SEF60 (*diamond*), and NCB226 (*inverted triangle*) sampled at flowering and mid-pod fill stage; mean values adjusted for treatment and development stage. Linear regression to calculate adjusted *R*^2^ and *P* values grouped according to treatment.

Carbon isotope abundance (δ^13^C) varied with treatment in the leaf tissue and seed with significant differences between WW and WD treatments and development stage (Figure 4 and Supplementary Table 5). Within the development stage, δ^13^C in leaf tissue significantly differs only in WW treatment (Figure 4A: HPWW–LPWW at flowering stage, *P* = 0.088; Figure 4B: HPWW–LPWW at mid pod fill, *P* = 0.013); while at harvest, δ^13^C in seed was substantially different under both WW (HPWW—LPWW, *P* = 0.000) and WD (HPWD—LPWD, *P* = 0.000) and becoming more depleted with WW conditions compared to WD (Figure 4C). Overall, there was significantly (*P* = 0.000) lower δ^13^C in leaf tissue at flowering (−28.65) and mid pod fill (−28.09) compared to seed at harvest (−25.35) (Figure 4 and Supplementary Table 5). No variation between breeding lines and commercial checks was detected for δ^13^C from the leaf or seed tissue (Supplementary Table 6). For the measured WW plants, the relationship between carbon and oxygen isotope abundance in the leaf tissue of select genotypes SEF60, NCB226, and DOR390 was weak (HPWW *R^2^* = −0.023, *P* = 0.445; LPWW *R^2^* = 0.196, *P* = 0.037) indicating the mild effect of LP treatment on the genotypes measured (Figure 4B). Albeit weak, a negative relationship between δ^13^C and δ^18^O indicates that the dominant influence on substomatal carbon concentrations is that of limitations to maximum net photosynthetic rate (*A*_*max*_), that is, a biochemical limitation to carboxylation imparted by low P availability rather than driven by changes in stomatal conductance.

### Impact of Low P and Water Deficit on Mineral Nutrients and Amino Acids in Leaf Tissue

**FIGURE 5:**
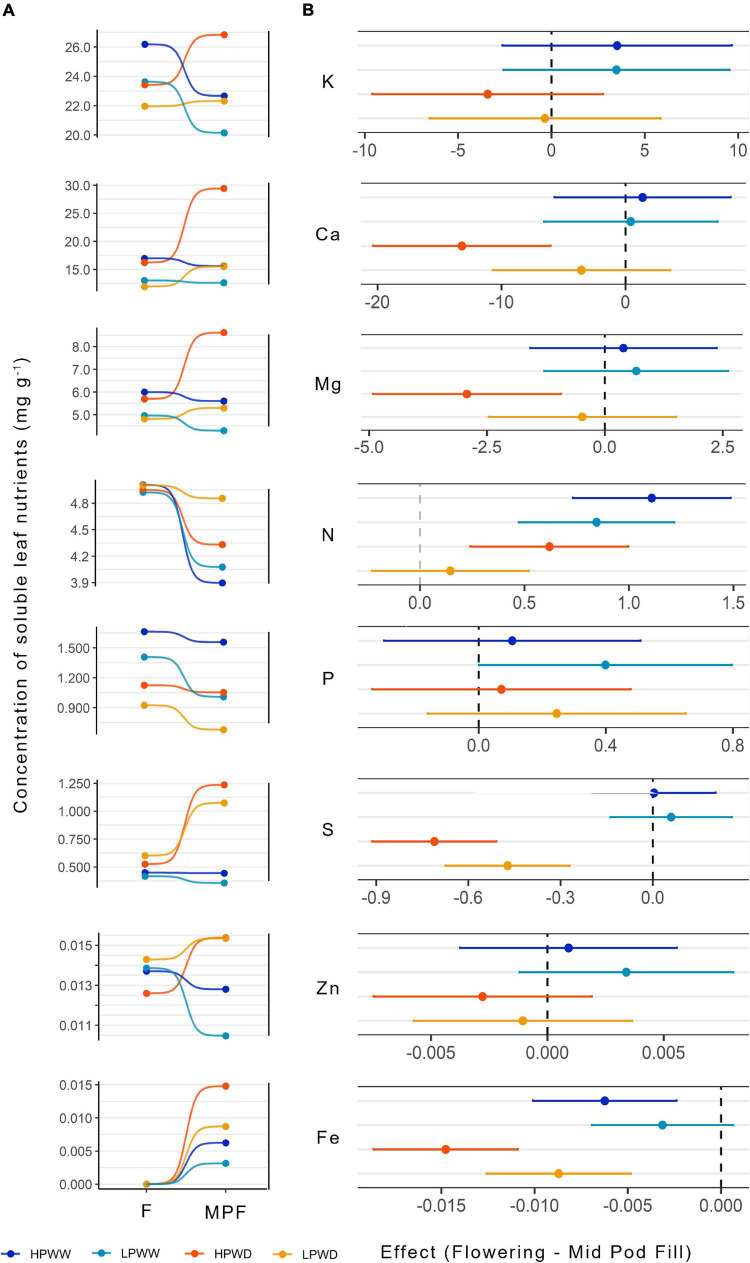
**(A)** Concentration (mg/g) of soluble leaf nutrients for 12 common bean lines under treatments of high phosphorus well-watered (HPWW; blue) low phosphorus well-watered (LPWW; light blue), high phosphorus water deficit (HPWD; orange), and low phosphorus water deficit (LPWD; yellow-orange) at flowering (F) and mid-pod fill (MPF) stage. **(B)** Effect of development stage (flowering—mid-pod fill) on the concentration of nutrients at different treatments. *Dots* represent mean value with upper and lower confidence level; *x-axis* shows the mean difference in the concentration of nutrients at four treatments; *vertical dash lines* illustrate 0 effect, where values close to 0 are not significantly different according to the HSD test at ɑ = 0.05. For visualization purposes, only four treatments are shown; the complete list of multiple treatments and stage comparisons is provided in Supplementary Table 7.

Within the soluble leaf extract, greater concentrations of potassium, calcium, and magnesium were present in comparison to other nutrients particularly iron and zinc (Figure 5). Variation in the concentration of minerals as a response to different treatments within the development stage was most evident at mid-pod fill. For example, there was significant treatment response on the concentration of phosphorus (HPWW—LPWD, *P* = 0.000) at flowering stage and calcium (HPWD—LPWW, *P* = 0.000), nitrogen (HPWW—LPWD, *P* = 0.000), and sulfur (HPWW—LPWD, *P* = 0.000) at mid-pod fill, among others (Figure 5A). In general, trends varied depending on the nutrient and concentration of minerals at the developmental stage (Figures 5A,B). The concentration of nitrogen (N) significantly declined from flowering to mid-pod fill stage for WW treatments regardless of level of P treatment (HPWW *P* = 0.000; LPWW *P* = 0.000). However, under WD treatment, there was no significant change from flowering and mid-pod fill at low P (LPWD, *P* = 0.935) but N slightly declined at high P treatment (HPWD, *P* = 0.000) (Figures 5A,B). The concentration of zinc significantly increased only at WD treatments at both P levels (HPWD and LPWD, *P* = 0.000) (Figures 5A,B). Strong differences between bred genotypes and commercial checks were only detected for zinc and iron in the soluble leaf tissue (Supplementary Table 8) while treatment responses do not seem to vary based on bred genotypes’ tolerance to drought or low P stress (see Table 1).

**FIGURE 6:**
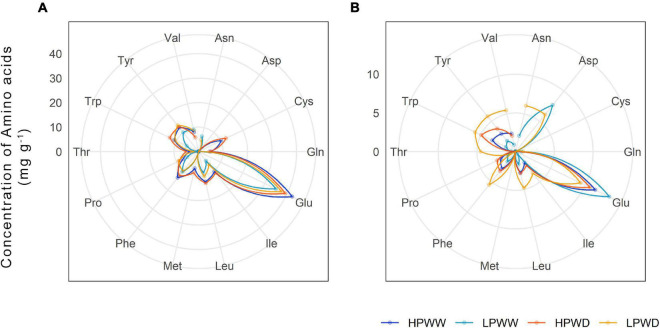
The concentration of amino acids (mg/g) found in common bean soluble leaf fraction subjected to high phosphorus well-watered (HPWW; blue), low phosphorus well-watered (LPWW; light blue), high phosphorus water deficit (HPWD; orange), and low phosphorus water deficit (LPWD; yellow-orange) at flowering **(A)** and mid-pod fill **(B)**. Data has been transformed for visualization purposes. Only Glu had a significant concentration level (*P* = 0.000) compared to other amino acids at all treatments at flowering and mid-pod fill.

In the soluble leaf tissue, significantly high concentrations of glutamic acid (Glu) were detected compared to all other amino acids (*P* = 0.000) at varying treatments and development stages. In addition, there was no significant change of Glu concentrations at both WW and WD and P concentrations (HPWW—LPWD, *P* = 0.177). However, Glu concentration significantly declined at mid-pod fill (*P* = 0.000) (Figure 6). There was a slight but not statistically significant increase in isoleucine, leucine, phenylalanine, threonine, tryptophan, and tyrosine in response to WD treatment (Figure 6). In general, concentrations of amino acids declined between flowering and mid-pod fill (*P* = 0.000) (Figure 6). Overall, there were no significant differences between bred genotypes and commercial checks across treatments and development stages, except for differences observed between low phosphorus and drought-tolerant–bred genotypes for Glu concentrations (Carioca—RCB593, *P* = 0.052).

### Impact of Low P and Water Deficit on Mineral Nutrients and Amino Acids in the Seed

**FIGURE 7:**
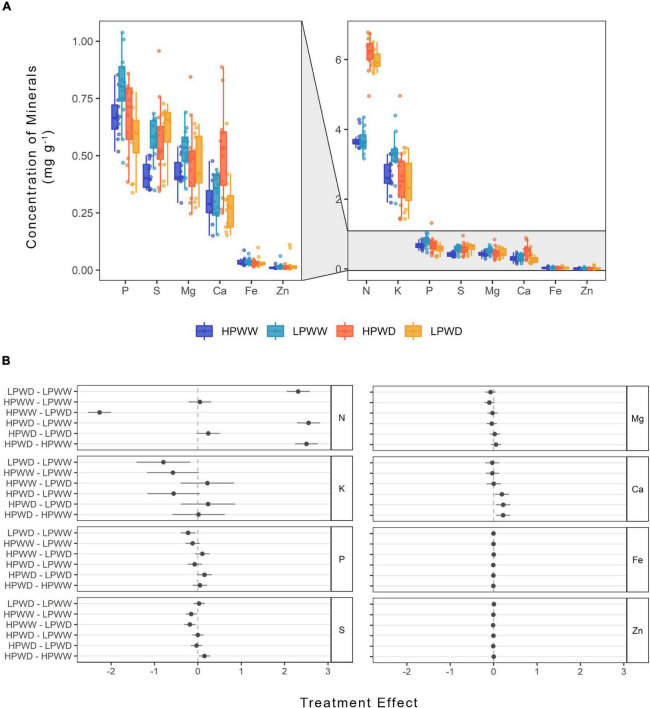
**(A)** Concentration (mg/g) of nutrients found in the common bean seed subjected to high phosphorus well-watered (HPWW; blue), low phosphorus well-watered (LPWW; light blue), high phosphorus water deficit (HPWD; orange), and low phosphorus water deficit (LPWD; yellow-orange) at harvest. Zoom in panel to the left shows nutrients that had concentrations less than 1 mg/g. **(B)** Comparison of treatment effect combination on the concentration of nutrients. *Dots* represent mean value with upper and lower confidence level; *x-axis* shows the mean difference in the concentration of nutrients between the treatments; *vertical dash lines* illustrate 0 effect, where values close to 0 are not significantly different according to the HSD test at ɑ = 0.05.

Nitrogen and potassium were present in higher concentrations in the digested total seed in comparison to other nutrients, particularly iron and zinc (Figure 7A). Significant treatment differences (*P* = 0.000), largely between WW and WD were observed for nitrogen, potassium, phosphorus, sulfur, and calcium (Figure 7B). Overall, there were no differences in the concentration of minerals between bred genotypes and commercial checks, except for nitrogen (Supplementary Table 9).

**FIGURE 8:**
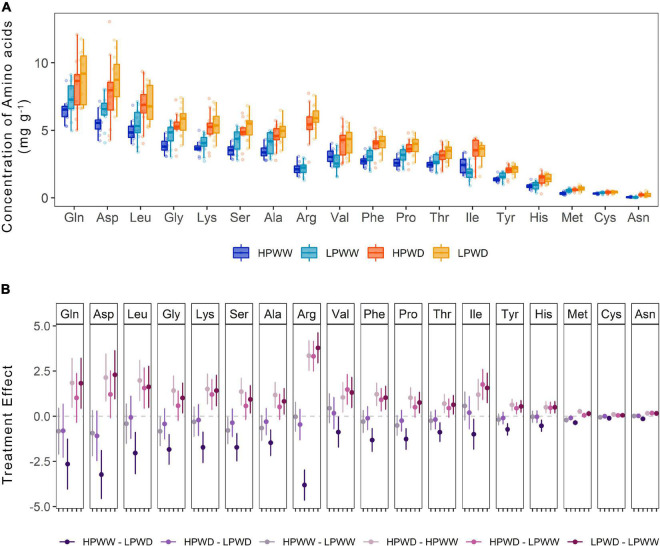
**(A)** Concentration of amino acids (mg/g) found in the common bean seed tissue subjected to high phosphorus well-watered (HPWW, blue), low phosphorus well-watered (LPWW; light blue), high phosphorus water deficit (HPWD; orange), and low phosphorus water deficit (LPWD; yellow-orange) at harvest. **(B)** Comparison of treatment effect combination on the concentration of amino acids. *Dots* represent mean value with upper and lower confidence level; *y-axis* shows the mean difference in the concentration of amino acids between the treatments; *horizontal dash lines* illustrate 0 effect, where values close to 0 are not significantly different according to the HSD test at ɑ = 0.05.

Almost all amino acids were detected in the seed tissue with the absence of only glutamic acid and tryptophan (Figure 8A). Statistically significant treatment differences were detected for the majority of amino acids as a consequence of the WD treatment under low P levels (HPWW—LPWD, *P* = 0.000) (Figure 8B). A comparison of genotype differences observed for any of the amino acids detected in the seed tissue is provided in Supplementary Table 9.

## Discussion

### Seed Yield and Aboveground Biomass Was Reduced Due to Individual and Combined Impacts of Water Deficit and Low P

Water stress and P deficiency, both individually and combined, significantly decreased seed weight and aboveground biomass by ∼80% (Figures 1, 2). This is unsurprising, given the substantial evidence for both abiotic stress’s influence on yield and its closely associated traits such as harvest index (Beebe et al., 2008; Smith et al., 2019). Yield loss was likely driven by reductions in aboveground biomass (i.e., less leaf area equals less photoassimilate), which in WD treatments led to a ∼75% reduction in the number of pods and seeds produced resulting in reduced yield (seed weight) (Figure 1). Leaf expansion is known to be highly sensitive to both water deficit and P deficiency (Rychter and Rao, 2005). There was a significant, but not dramatic, reduction in remobilization, as indicated by lower values of pod harvest index, in WD plants compared to those in the WW treatment (Figure 1). Within the combined LPWD treatment, the pod harvest index was significantly lower in comparison to the combined HPWD treatment (Figure 1). PCA also showed the differing response of pod harvest index compared to other yield parameters suggesting that the trait is influenced by other factors beyond those measured (Figure 1A). This response also suggests that water and P availability play an important role in the movement of photoassimilate from the pod wall into the developing seed. Remobilization of photosynthate to seed (indicated here by measures of pod harvest index) is known to play a significant role in improved adaptation to drought and low P in common bean (Beebe et al., 2008, 2009; Assefa et al., 2013; Rao et al., 2013). However, the mechanisms that determine the movement of photoassimilate from the pod wall and into the seed are not well understood and further research is required to characterize the underlying causes of poor remobilization often described as, “lazy pod syndrome” (Beebe, 2012). More broadly, variation in seed weight and aboveground biomass associated with genotype was observed among the 12 common bean genotypes investigated (Figure 2). The variation in harvest index (Figure 1C) between the genotypes bred for adaptation to drought and low P availability and the commercial checks highlights the positive impact of breeding activities on yield. Nevertheless, further investigation is warranted to determine the mechanistic relationships of abiotic stress factors such as water deficit and P deficiency on the assimilation and transport of resources throughout yield development and the impacts this combined stress has on yield.

### Water and P Deficiencies Influence Carbon Assimilation and Water Use at the Leaf and Plant Scale

Water and P deficiency significantly decreased photosynthesis and stomatal conductance over plant development (Figure 3). In this study, for plants grown under LPWD, aboveground biomass was significantly lower (Figure 2) which subsequently reduced whole-plant water use, therefore, decoupling plant growth from the treatment. For example, this may have allowed for the maintenance of photosynthesis at similar rates per unit leaf area as plants grown under the HPWD treatment which had a slightly higher aboveground biomass and hence would have required greater amounts of water at the whole plant scale (Figures 2, 3). This response indicates the importance of P supply on whole-plant function *via* the regulation of source–sink dynamics (Rychter and Rao, 2005; Smith et al., 2018b).

Genotypic variation in photosynthesis and stomatal conductance was detected for all genotypes measured (Figure 3) and has also been observed in previous studies using common bean (Wentworth et al., 2006; Polania et al., 2016c; Rao et al., 2017). For the three genotypes, NCB226, SEF60, and DOR390, selected for *A/c_*i*_* curves under the LPWW and HPWW treatments, significant treatment and genotypic differences in the biochemistry that underlies photosynthesis were only detected in the flowering stage (see Supplementary Figure 1). We expected greater treatment differences in biochemistry as a consequence of P deficiency due to the important role P plays in the Calvin Cycle reactions (Raines, 2003). This response highlights that the LP treatment only caused mild deficiency (also indicated by the weak negative correlations between δ^13^C and δ^18^O, Figure 4D) while the WD increased in severity as development continued. Understanding how photosynthesis, along with the underlying biochemistry, responds to multiple stresses and fluctuating environmental conditions over time is critical (see, e.g., Zhu et al., 2010).

### Isotope Abundance Indicates Drought Stress in Leaf and Seed

Carbon isotope fractionation in the leaf tissue significantly decreased by approximately 2 parts per million in response to the WD treatment at all development stages (Figure 4A) while in the seed tissue collected at harvest, significant differences were observed between all treatments with decreases due to HP and WD treatments (individually and combined) (Figure 4A). These responses are similar to those detected in previous studies (Badeck et al., 2005; Smith et al., 2016, 2018a, 2019). The difference in the responses between the leaf and seed likely occurred due to heterotrophic fractionation, which may occur in part as a consequence of changing demand for metabolites in sink tissues altering the carbon isotope abundance of the remaining soluble pool (see Cernusak et al., 2009; Smith et al., 2018a). This can be magnified by changes in environmental conditions (Smith et al., 2016) and in this case, resulted in the carbon isotope abundance of the seed harvested from the LP treatment significantly differing from other treatments, while in the leaf tissue the P treatment had no significant impact.

While carbon isotope abundance indicates drought stress, it does not necessarily equate directly to measures of water deficit or water use efficiency (see Seibt et al., 2008). Nevertheless, carbon isotope abundance has been proposed and utilized as a screening method to improve water use efficiency in breeding C3 crops, including that of common bean (Polania et al., 2016c). Using carbon isotope abundance from seed, common bean genotypes have previously been classified as “water spenders” and “water savers” (Polania et al., 2016c), however, in this case, no genotypic differences were detected in either leaf or seed tissue under any treatment conditions. Relationships between δ^13^C and growth are yet to be fully described. Consideration must be made of the source of carbon for analysis, and the likelihood of the isotope abundance contained within it to reflect water use efficiency across realistic time and spatial scales. The present study did not uncover a strong relationship between δ^13^C and δ^18^O as expected (see Scheidegger et al., 2000; Figure 4B) perhaps due—at least in part—to the relatively isohydric response of common bean to such a severe water deficit. During periods of severe water deficit, changes in isotope signals are reduced because of very low gas exchange and little net assimilation of carbon. The present study highlights that the application and interpretation of carbon isotope abundance has limits imposed by plant responses at the boundaries of physiological function.

### Leaf Mineral Nutrient and Amino Acid Concentrations Decline in Response to Water and P Deficiency

Within the soluble leaf tissue, concentrations of mineral nutrients and amino acids declined in response to both the individual and combined effects of WD and LP (Figures 5, 6). The majority of the mineral nutrients detected within the soluble leaf fraction at flowering and mid-pod fill decreased in response to reductions in P availability (Figures 5A,B). Significant variation between genotypes was detected for zinc and iron, which according to the statistical model significantly interacted with genotype and treatment (Supplementary Table 7). This may have been a consequence of mobilization of iron, as this has not influenced the concentration of iron present in the seed, with similar concentrations of iron found under all treatments and no significant differences between genotypes (Figure 7B). This finding contrasts with that of Petry et al. (2015) who reported that location and management can alter iron and zinc seed concentration; however, these findings are similar to those reported by Bulyaba et al. (2020) who found that iron and zinc concentration in the seed did not significantly vary between locations with varying soil properties or varieties. While it is known that leaves serve as a substantial source of mineral nutrients for developing seeds (Garcia and Grusak, 2015), a few studies outside the initial work of Hocking and Pate (1977) have researched concurrent changes in leaf and seed mineral content (Garcia and Grusak, 2015). Seed nutrient quality is likely regulated in part by source–sink dynamics and as such the manipulation of transport processes between the leaf tissue toward the developing reproductive tissue is a possible strategy to increase nutrient allocation within the seed (Bennett et al., 2011; Pottier et al., 2014; Garcia and Grusak, 2015; Tan et al., 2017; Smith et al., 2018b).

The concentration of most amino acids detected significantly differed with WD (Figure 6). Variation between genotypes was detected for amino acids at the leaf level (data not shown). In particular, the proportion of amino acids detected showed that in some genotypes, very few amino acids (aspartic acid and glutamic acid) were present in the soluble leaf fraction. The examination of the soluble leaf fraction has previously been used to infer stress responses of plants (see, e.g., Hare et al., 1998; Merchant et al., 2010; Dumschott et al., 2017). Given the variation in nutritional quality (mineral nutrients and amino acids) between each of the genotypes, further investigation may allow for the targeted improvement of the genotypes by promoting metabolites that confer resilience. While this type of monitoring could be used to reduce some of the genotypic variations and increase yield, no genotypic differences were detected for nutritional quality in the seed (Figures 7, 8 and Supplementary Table 9), and as such amelioration of reductions to nutritional quality in the seed under varying abiotic stress conditions is expected to be difficult.

### Water and P Deficit Impacted Nutritional Quality Equally Among Genotypes

Water stress and P deficiency, both individually and combined, significantly decreased the concentration of most mineral nutrients detected (potassium, phosphorus, sulfur, calcium) in the seed (Figure 7). However, under WD conditions nitrogen concentration in the seed was found to increase by ∼50% and slightly further under the HPWD treatment (Figures 7A,B). This may be a consequence of increasing rates of remobilization, a well-documented response under abiotic stress conditions in common bean (Assefa et al., 2013; Beebe et al., 2013; Rao et al., 2013; Polania et al., 2016b,c). The increase in the nitrogen pool of the seed is reflected in the number of individual amino acids (Figure 8). Statistically significant increases (of between ∼2 and 50%) in the concentrations of most amino acids detected (alanine, arginine, asparagine, histidine, leucine, lysine, methionine, phenylalanine, proline, serine, threonine, tyrosine, valine) were found in the total seed in response to WD (Figure 8B). These results are in agreement with Gyori et al. (1998) who detected an increase in amino acids as a consequence of water stress in common bean and Nayyar et al. (2006) and Behboudian et al. (2001) who detected a similar increase of amino acids, and similar decrease of mineral nutrients, under water stress in chickpea (*Cicer arietinum* L.). Despite the relative importance of stress on the nutritive value of seeds, the mechanisms of accumulation of amino acids in the seed under stress are not well understood (Behboudian et al., 2001). Given that little attention has been directed toward the improvement of protein concentration in common beans in recent years as a result of negative correlations between protein concentration and seed yield (Beebe, 2012), further investigation into the accumulation of protein compounds under abiotic stress is required.

Nutritional quantity per seed or concentration (mineral nutrients and amino acids) did not vary in any of the genotypes measured. The lack of genotypic variation in nutritional content demonstrates that current breeding activities have maintained seed nutrient content notwithstanding successful efforts to increase seed yield quantity (Beebe et al., 2008). For instance, in this study, while superior-bred genotypes such as NCB226 maintained higher seed yield under WD and LP, compared to a commercial check such as DOR390 (Figure 1), and leaf-level nutrient concentration varied between the two genotypes (Figure 5), there were no statistically significant differences in the concentration of mineral nutrients and amino acids detected within the seed at harvest. The processes that have maintained seed nutrient concentration despite genotypic variation are not entirely clear. One hypothesis is that nutritional content may have been preserved due to the evolutionary requirements for seed germination. Given that a germinating seed requires a minimum amount of nutrients for successful germination and growth (see, e.g., White and Veneklaas, 2012) it is intuitive that the seed would reflect this need. Nevertheless, genotypic variation for nutrient content in common bean is present in the germplasm (Beebe et al., 2000; Blair et al., 2011; Blair, 2013) and has been exploited to increase the concentration of iron and zinc in common bean seed of recently released biofortified cultivars in Africa and Latin America (Haas et al., 2016; Andersson et al., 2017).

This investigation has demonstrated that water stress and P deficiency have substantial impacts on yield and yield-related parameters including aboveground biomass. Water deficit and low P led to physiological changes at the leaf level, including reductions to leaf-level photosynthesis and stomatal conductance, changes to carbon isotope abundance, and genotypic variation in the soluble pool of mineral nutrients and amino acids. Water deficit significantly increased the concentration of mineral nutrients and amino acids in the seed although no genotypic variation was detected, likely as a result of the need for viable seeds for germination. Plants produce seeds, not for human nutrition but to sustain embryonic development at germination. Enhancing our knowledge of seed development and its resilience under realistic, natural environments containing multiple stresses has great potential to provide more informed management and breeding activities to improve the nutritional value and quantity of yield production.

## Data Availability Statement

The datasets presented in this study can be found in online repositories. The names of the repository/repositories and accession number(s) can be found in the article/Supplementary Material.

## Author Contributions

MS, EV, JP, IR, SB, and AM conceived and designed the experiment. MS, EV, JP, and IR performed data collection and site management. ED produced the data analysis and figures. MS and AM wrote the original manuscript with input from ED, EV, IR, and JP. All authors contributed to the article and approved the submitted version.

## Conflict of Interest

The authors declare that the research was conducted in the absence of any commercial or financial relationships that could be construed as a potential conflict of interest.

## Publisher’s Note

All claims expressed in this article are solely those of the authors and do not necessarily represent those of their affiliated organizations, or those of the publisher, the editors and the reviewers. Any product that may be evaluated in this article, or claim that may be made by its manufacturer, is not guaranteed or endorsed by the publisher.

## Supplementary Material

Supplementary Figure 1 *A/c_*i*_* parameters *V*_*cmax*_ (μmol/m^2^/s), *J* (μmol/m^2^/s) and TPU for common bean lines DOR390, SEF60, and NCB226 under treatments of high phosphorus well-watered (HPWW; blue) and low phosphorus well-watered (LPWW; light blue) at flowering.
