# Supplementary figures and images for: Effect of Drought and Low P on Yield and Nutritional Content in Common Bean
Source: Front Plant Sci. 2022 Mar 29;13:814325. doi: 10.3389/fpls.2022.814325 (PMC9002355; doi:10.3389/fpls.2022.814325)

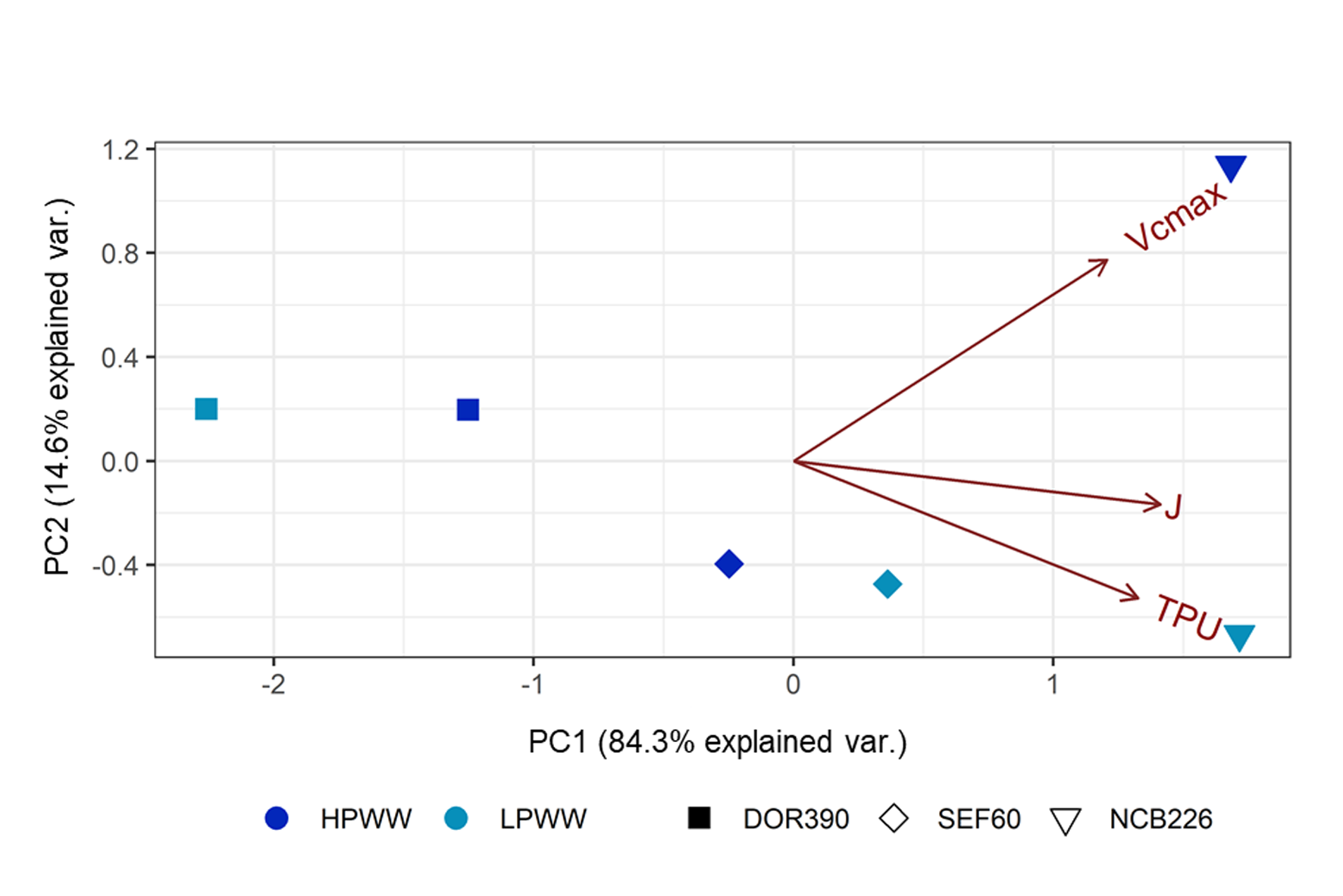

Supplement: Supplementary Figure 1 — A/ci parameters Vcmax (μmol/m2/s), J (μmol/m2/s) and TPU for common bean lines DOR390, SEF60, and NCB226 under treatments of high phosphorus well-watered (HPWW; blue) and low phosphorus well-watered (LPWW; light blue) at flowering. [file Image_1.TIF]
